# Air-Chambers

**Published:** 1863-08

**Authors:** J. D. Anderson

**Affiliations:** Sacramento, California


### AIR-CHAMBERS.

BY J. D. ANDERSON.

A few years ago a great deal was said and written on the above subject, and, until I read the March number of the *Dental Cosmos*, I had supposed that the profession had come to the wise conclusion that much time and labor had been wasted in constructing these useless cavities which only serve to produce a bunch of turgid membrane, which reminds us that "all proud flesh, wherever it grows, is subject to irritation." And when I read Dr. Way's recommendation to substitute two chambers for one (in the *Cosmos* above referred to) it occurred to me that *that way* more than doubles the difficulty. But I have a plan which is simple and effective and think I shall never find it necessary to adopt any other. It has long been a settled fact that when two substances fit *perfectly* together that atmospheric pressure (being about fifteen pounds to the square inch) will *hold* them together; and this principle will apply to metallic plates and "boneless gums" as well as to the two blocks of marble the old Professor always alludes to. And this being so, it is only necessary to adapt the plate perfectly to the part it is designed to cover and it will adhere so firmly that no movement of the lips or muscles can possibly displace it. There is usually, however, a ridge along the mesial line of the palate that is much harder than the rest of the arch, and unless something is done to obviate the difficulty the plate will rock and its adhesion be endangered. This is particularly unfortunate for Dr. Way's plan of making an air-chamber on each side of the mesial line as the "division between the holes," which, he claims, prevents the filling up of the two cavities, rests directly on this unyielding ridge in the centre of the palate. The best plan, I think, is to take some thin substance (say a business card) cut it to fit over the centre of the palate and swage the plate over it. This is not for the purpose of making an air-chamber but simply of relieving the stubborn ridge so that it cannot rock the plate out of its position. If this precaution is taken and the plate fits well it will cling to its place with the tenacity that man clings to life, each being supported by the same compound—atmospheric air.

SACRAMENTO, CALIFORNIA, May 16, 1863.

